# Evaluation of water quality and human risk assessment due to heavy metals in groundwater around Muledane area of Vhembe District, Limpopo Province, South Africa

**DOI:** 10.1186/s13065-017-0369-y

**Published:** 2018-01-12

**Authors:** Joshua Nosa Edokpayi, Abimbola Motunrayo Enitan, Ntwanano Mutileni, John Ogony Odiyo

**Affiliations:** Department of Hydrology and Water Resources, University of Venda, Private Bag X5050, Thohoyandou, 0950 South Africa

**Keywords:** Contamination, Groundwater, Health risk, Multivariate analysis, South Africa

## Abstract

Groundwater is considered as good alternative to potable water because of its low turbidity and perceived low contamination. The study assessed the physio-chemical and heavy metals concentrations in eight randomly selected boreholes water at Muledane village in Limpopo Province of South Africa and the results were compared with South African National standard permissible limit. The impacts of heavy metals on human health was further determined by performing quantitative risk assessment through ingestion and dermal adsorption of heavy metals separately for adults and children in order to estimate the magnitude of heavy metals in the borehole samples. Parameters such as turbidity, nitrate, iron, manganese and chromium in some investigated boreholes did not comply with standard limits sets for domestic water use. Multivariate analyses using principal component analysis and hierarchical cluster analysis revealed natural and anthropogenic activities as sources of heavy metal contamination in the borehole water samples. The calculated non-carcinogenic effects using hazard quotient toxicity potential, cumulative hazard index and chronic daily intake of groundwater through ingestion and dermal adsorption pathways were less than a unity, which showed that consumption of the water could pose little or no significant health risk. However, maximum estimated values for an individual exceeded the risk limit of 10^−6^ and 10^−4^ with the highest estimated carcinogenic exposure risk (CR_ing_) for Cr and Pb in the groundwater. This could pose potential health risk to both adults and children in the investigated area. Therefore, precaution needs to be taken to avoid potential CR_ing_ of people in Muledane area especially, children using the borehole water. 
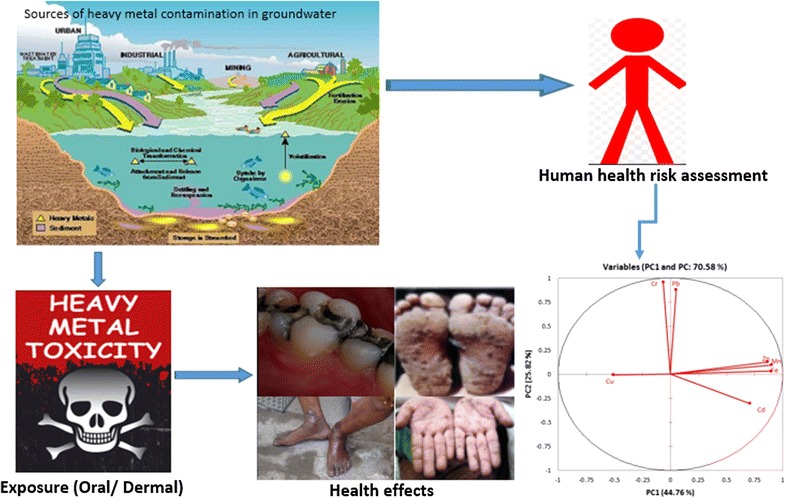

## Introduction

Sustainable access to potable water have been achieved in different developed countries of the world, but this is not true for many developing countries. In Africa, access to potable water has been achieved in a few cities but not in the entire region. This problem is more pronounced in rural areas, some of which does not have water supply infrastructure [1]. Residents of such rural communities often resort to different sources of water. The most commonly used sources include: Rivers, streams, boreholes, lakes, etc. Most of these various alternative sources are susceptible to water pollution. Some of the major sources of pollution include the discharge of domestic, industrial and agricultural wastewater into freshwater bodies.

Groundwater is often considered as the best of these alternatives, owing to natural protection from pollution when compared to surface and perceived natural filtration as water flows down during rainy period. Groundwater as one of the natural resources is of fundamental importance to human life, because of its perceived good microbiological quality in the natural state and as a result, it is often the preferred source of drinking water supply as treatment is limited to disinfection. Aesthetically, it looks clean and acceptable to various people as it is often free from odour and sometimes do have a pleasant taste. Despite the perceived safety associated with groundwater consumption, several researches have shown that groundwater can also be susceptible to contamination [2–4]. Some factors that influence the quality of groundwater include the geology of the aquifer, climate and anthropogenic activities [5–8].

The use of groundwater sources has increased rapidly in many countries of the world due to population growth, increased industrialization and scarcity of water related to climate change. Although surface water has been extensively used in various water infrastructure, increased utilization coupled with other aforementioned factors has led to an increase in the use of groundwater sources. Groundwater are often used for drinking, irrigation and several industrial processes. The global use of groundwater is often underestimated and climatic factor has also been extensively debated to influence the available water volume in the aquifer [9]. Several countries of the world are experiencing acute water scarcity, but this problem is exacerbated in arid and semi-arid countries of the world. The use of shallow, such as hand dug wells and deep groundwater sources (boreholes) are common in South Africa. Most of the communities that depends on groundwater sources do not know the quality of water they drink as they often presume that groundwater has a good water quality. Groundwater can be contaminated by the ingress of human and animal waste into the aquifer [10]. This could be through the grazing of animals, discharge of domestic and industrial wastewater, use of pesticides and fertilizers in agriculture [11].

In some part of South Africa, groundwater is a key component of the water resources, and one of the sources of water supply. Report have shown that about two-thirds of South African population depend on groundwater for drinking [12, 13] with about 65% of the total supply in the rural areas [14]. As such, it provides some basic water requirement, since the country’s surface water resources are unevenly distributed and cannot meet the growing demand for water [15]. In rural areas, boreholes are located either close to a pit toilet or downstream of soak away pits or adjoining landfills or dumpsites [16]. Some groundwater is poorly managed due to its invisible nature and it usually takes a long time to notice when it has become polluted and once it is contaminated, its quality cannot be restored by just stopping the pollutants from source, because contamination may continue after the source has been stopped or removed [17, 18]. In the rural and peri-urban areas, most of the groundwater supplies are usually untreated and it has been reported that it is difficult for groundwater to purify itself, often impossible and very expensive to treat, thereafter [14]. The use of groundwater sources of unknown quality puts the consumers at risk to possible waterborne diseases. Bessong et al. [19] reported high levels of fecal contamination in groundwater sources around Tshikuwi Community in Vhembe District of South Africa. High fluoride levels have been reported by Odiyo and Makungo [20] in groundwater sources around Siloam village. Arsenic contamination of groundwater sources has been reported in the world [2, 21].

Thohoyandou, Vhembe District of Limpopo, South Africa is experiencing a rapid population growth and this has led to an increase in the generation of waste. Muledane village in Thohoyandou consist of households that rely on groundwater while, some areas are reserved for municipal landfill site, farming, wastewater treatment plant and cemeteries. Landfills have been identified as one of the major threats to groundwater resources in this area [22]. There is currently no published data on the status of groundwater quality in Muledane village and possible health risks that these water sources may have on humans, unlike other reports of groundwater quality in South Africa that reported the impact of heavy metals, physical and chemical properties on human health [23, 24]. Hence, there is an urgent need to assess water quality of groundwater in Muledane village because contaminated water by faeces, leachate and other non-point sources could have economic and social development implications and human health risks due to activities around this area. It is assumed that water quality impairment might be severe in Muledane village of Thohoyandou. To this end, the aim of this study was to assess the status of water quality from boreholes situated at Muledane area near Thohoyandou by quantifying heavy metal concentration and determine possible health risk due to exposure of human to heavy metals.

## Materials and methods

### Study area and land use

The study area is located at Thohoyandou block J in Thulamela Municipality Government area of Vhembe District, Limpopo province. Geological coordinates of Muledane area is located approximately on longitudes 30°1′0″E and latitudes 23°29′0″N, respectively at 734 m elevation above the sea level. The Thulamela municipality area is approximately 2966, 4 km in extent which covers 13, 86% of the total area of the Vhembe District with an estimated population of 537,454 [25]. Activities around Muledane area consist of schools, churches, agricultural activities, residential and hotels. It also encompasses dense bushes and trees, sewage treatment plant and the municipal landfill site which make up a large portion of the study area. Thohoyandou falls under the summer climatic conditions of South Africa with very warm conditions and the annual rainfall ranging from 400 to 800 mm. Rainfall during summer is very high with little rainfall in winter. The temperatures may reach up to 37 and 23 °C on the average in summer and winter, respectively [26]. The 1:1,000,000 scale geological map of South Africa from the council for Geoscience shows that Muledane is dominated by fractured aquifers [27]. The depth of water table derived from National Groundwater Database (NGDB) range from 15 to 30 m. The recharge map compiled by DWAF as part of the Groundwater Resources Assessment study of 2004 indicate that Muledane range from 10 to 50 mm/annum [28].

### Sample collection, preparation and storage

Groundwater samples were collected as outlined by Fitfield and Haines [29]. Briefly, plastic bottles were washed and stored in 10% nitric acid for 2 days and rinsed with double distilled water before sampling. A total of 24 groundwater samples were collected from eight randomly selected boreholes at Muledane area of Thohoyandou. Borehole samples were label according to their sources using the code B1–B8. The bottles were rinsed three times and taps were allowed to run for at least 5 min before collection of samples and labelled accordingly. Samples for metals were preserved by adding 3 mL of concentrated HNO_3_. All the samples were placed on an ice chest and transported to the University of Venda then preserved at − 4 °C in the refrigerator for further analysis.

### Analytical methods

Onsite analysis of the physico-chemical parameters such as electrical conductivity (EC) and turbidity were measured on-site using Cyberscan 500 conductivity meter (AQ2010 LABOTEC) and turbidity meter, respectively. The pH and temperature were measured using pH meter (H1 8014 HANNA instrument). Appropriate portion of the collected groundwater samples were digested with concentrated HNO_3_ for heavy metals analysis according to the method of Sharma [30] and analysed using an inductively coupled plasma optical atomic spectrophotometer (ICP-OES) (ThermoScientific). The instrument was standardized with seven working standard solutions (multi-point linear fitting) for Copper (Cu), Manganese (Mn), Iron (Fe), Chromium (Cr), Cadmium (Cd), Zinc (Zn) and Lead (Pb) and analytical precession was checked by frequently analysing the standards as well as blanks. An Ion Chromatography (Methrohm 850 Professional IC) was used to analyze the anions concentration including nitrates, chlorine, fluorine, and sulphates in water samples collected from different boreholes so as to check the groundwater’s suitability for domestic use. The IC has 20 μL injection loop, Ionpac AG144× 50 mm guard and AS144× 250 mm analytical columns with conductivity detector. Multiple working solutions of 1, 5, 10 and 20 units/ppm were prepared and used in calibrating each anion Fluoride (F^−^), Chloride (Cl^−^), Nitrate (NO_3_^−^) and Sulphate (SO_4_^2−^). An eluent 1.0 Mm NaHCO_3_/3.5 Mm Na_2_CO_3_ was prepared and pumped through the IC system. The standards were injected into the instrument sequentially, in order to perform calibration for each element. The samples were filtered through a 0.45 μm Millipore filter and then injected into IC machine for analysis.

### Quantitative health risk assessment

Human exposure risk pathways of an individual to trace metals contamination could be through three main pathways including inhalation via nose and mouth, direct ingestion and dermal absorption through skin exposure. Common exposure pathways to water are dermal absorption and ingestion routes. Exposure dose for determining human health risk through these two pathways have been described in the literature [31–33] and can be calculated using Eqs. 1 and 2 as adapted from the US EPA risk assessment guidance for superfund (RAGS) methodology [31, 33].

1 $$ Exp_{ing} = \frac{{C_{water} \times \;IR\; \times \;EF \; \times \;ED}}{BW \; \times \;AT} $$

2 $$ Exp_{derm} = \frac{{\left( {C_{water} \; \times \;SA\; \times \;KP\; \times \;ET\; \times \;EF\; \times \;ED \; \times \;CF} \right)}}{{\left( {BW \times AT} \right)}} $$

where, Exp_ing_: exposure dose through ingestion of water (mg/kg/day); Exp_derm_: exposure dose through dermal absorption (mg/kg/day); C_water_: average concentration of the estimated metals in water (μg/L); IR: ingestion rate in this study (2.2 L/day for adults; 1.8 L/day for children); EF: exposure frequency (365 days/year); ED: exposure duration (70 years for adults; and 6 years for children); BW: average body weight (70 kg for adults; 15 kg for children); AT: averaging time (365 days/year × 70 years for an adult; 365 days/year × 6 years for a child); SA: exposed skin area (18,000 cm^2^ for adults; 6600 cm^2^ for children); Kp: dermal permeability coefficient in water, (cm/h), 0.001 for Cu, Mn, Fe and Cd, while 0.0006 for Zn; 0.002 for Cr and 0.004 for Pb [34]; ET: exposure time (0.58 h/day for adults; 1 h/day for children) and CF: unit conversion factor (0.001 L/cm^3^) [31–33, 35].

Potential non-carcinogenic risks due to exposure of heavy metals were determined by comparing the calculated contaminant exposures from each exposure route (ingestion and dermal) with the reference dose (R*f*D) [31] using Eq. 3 in order to develop hazard quotient (HQ) toxicity potential of an average daily intake to reference dose for an individual via the two pathways using Eq. 4.

3 $$ HQ_{ing/derm} = \frac{{Exp_{ing/derm} }}{{RfD_{ing/derm} }} $$

where RfD_ing/derm_ is ingestion/dermal toxicity reference dose (mg/kg/day). The RfD_ing_ and RfD_derm_ values were obtained from the literature [31–33, 35, 36]. An HQ under 1 is assumed to be safe and taken as significant non-carcinogenic [37], but HQ value above 1 may be a major potential health concern in association with overexposure of humans to the contaminants.

To assess the overall potential non-carcinogenic effects posed by more than one metal and pathway, the sum of the computed HQs across metals was expressed as hazard index (HI) using Eq. 4 [31]. HI > 1 showed that exposure to the groundwater could have a potential adverse effect on human health [32, 34].

4 $$ HI = \mathop \sum \limits_{i = 1}^{n} HQ_{ing/derm} $$

where HI_ing/derm_ is hazard index via ingestion or dermal contact. Chronic daily intake (CDI) of heavy metals through ingestion was calculated using Eq. 5;

5 $$ CDI = C_{water} \times \frac{DI}{BW} $$

where C_water_, DI and BW represent the concentration of trace metal in water in (mg/kg), average daily intake of water (2.2 L/day for adults; 1.8 L/day for children) and body weight (70 kg for adults; 15 kg for children), respectively. Carcinogenic risk (CR) through ingestion pathway was estimated using Eq. 6:

6 $$ CR_{ing} = \frac{{Exp_{ing} }}{{SF_{ing} }} $$

where, CR_ing_ is the carcinogenic risk via ingestion route and SF_ing_ is the carcinogenic slope factor where Pb is 8.5E, Cd is 6.1E+03 and Cr is 5.0E+02 µg/kg/day [33, 34, 36]. The CR_ing_ values for other metals were not calculated due to unavailability of the SF_ing_ values.

### Statistical analysis

GraphPad Prism version 5.0 for Windows (GraphPad Software, San Diego California, USA) was used for both statistical analysis at 95% confidence limit and the graphs. Mean values of the parameters obtained for the various locations were compared to DWAF [38] and WHO [39] guidelines for domestic water use. Multivariate statistics in terms of principal component analysis (PCA)/factorial analysis (FA) and hierarchical agglomerative analysis (HAC) were performed using Xlstart statistical software [40]. The PCA is used to established major variation and relationships among the different metals. Pearson correlation was calculated for different metals in groundwater samples and significant principal components (PC) was selected based on the varimax orthogonal rotation with Kaiser normalization at eigenvalues greater than one. The HCA was used to identify groups that shows similar characteristics or variables and dendrogram to provide a visual summary of the results based on dimensionality of the original data [34].

## Results and discussion

Table 1 shows the turbidity, temperature, pH, conductivity and TDS of groundwater samples collected from Muledane village. The pH varied from slightly acidic to neutral (6.04–7.41) throughout the sampling period. These values were within the recommended guideline of DWAF (6.0–9.0) for domestic water use [38]. The pH values for all borehole except for B2 was higher in the months of January as compared to other months. This is not expected because the pH of rainwater is low and could influence groundwater’s pH due to high infiltration of aquifer during heavy rainfall. The acidity or alkalinity of water can affect plant growth, benthic organisms, soil and crops when used for irrigation. This could also indicate possible corrosion problems and potential heavy metals contamination. Copper, Zn and Cd are associated with low values of pH, e.g., a pH of 2 will cause water to be acidic and unsuitable for human consumption [41].

**Table 1:** Mean value of physico-chemical parameters in groundwater samples collected from eight boreholes in Muledane village

Settlement	Range of sample stations	Month of sampling	Turbidity (NTU)	pH	EC (mS/cm)	TDS	F^−^	Cl^−^	NO_3_^−^	SO_4_
B1	30°28′24.1″E 23°00′16.7″S	January	0.65	7.17	61.90	414.73	0.17	30.07	121.64	15.25
		April	0.99	6.51	63.70	426.79	0.06	23.93	53.13	12.55
		June	1.16	6.96	64.00	428.8	0.12	32.42	51.55	13.90
B2	30°28′13.5″E 23°00′09.4″S	January	1.12	6.75	40.90	274.07	0.09	41.89	14.23	1.76
		April	0.54	6.22	42.90	287.43	0.01	35.85	5.81	1.63
		June	0.94	6.79	43.70	292.79	0.05	46.47	57.38	1.70
B3	30°27′19.0″E 22°59′43.1″S	January	4.10	6.82	32.90	220.43	0.09	28.87	30.49	2.03
		April	14.9	6.22	18.68	125.16	0.09	14.22	11.97	1.61
		June	1.28	6.39	20.18	135.21	0.09	16.80	11.84	1.82
B4	30°27′17.8″E 22°59′43.1″S	January	1.22	7.19	4.48	29.49	0.04	4.30	0.70	0.97
		April	3.50	6.17	31.90	213.73	0.04	46.91	0.60	0.80
		June	1.11	7.20	16.28	109.08	0.04	42.61	0.65	0.97
B5	30°27′15.5″E 22°59′43.0″S	January	1.00	6.95	11.30	75.71	0.04	10.14	16.08	0.65
		April	5.76	6.13	16.29	109.14	0.017	11.29	2.2	0.61
		June	1.04	6.71	19.47	130.45	0.02	14.92	9.16	0.63
B6	30°27′14.8″E 22°59′42.2″S	January	1.38	6.76	12.70	85.09	0.05	11.10	16.37	1.22
		April	1.62	6.22	8.99	60.23	0.07	2.67	0.85	0.81
		June	1.16	6.28	9.78	64.99	0.087	8.45	72.71	1.01
B7	30°27′13.9″E 22°59′44.2″S	January	2.26	6.62	28.20	188.94	0.047	19.92	47.86	4.87
		April	0.94	6.04	15.11	101.24	0.047	2.53	5.03	0.76
		June	1.66	6.57	9.81	65.73	0.047	12.60	2.25	2.81
B8	30°27′12.0″E 22°59′47.8″S	January	0.33	6.98	61.30	410.71	0.16	30.70	125.18	16.14
		April	0.79	6.49	40.90	274.03	0.16	34.18	63.73	14.56
		June	1.01	6.59	51.10	342.37	0.16	3.48	2.27	16.14
Standard limit for WHO for drinking water quality			1.0	6.0–9.0	≤ 70	≤ 450	1.0	100	50	< 200
Standard limit for DWAF for drinking water quality			< 5	–	–	< 1000	< 1.5	–	< 22	–

Concentration are in mg/L except otherwise stated

The EC average level for each sampling point during the monitoring period were 63.2, 42.5, 23.92, 17.56, 15.69, 10.52, 17.71 and 51.1 mS/cm for samples B1–B8, respectively. The mean values recorded for conductivity were within the recommended guideline of < 70 mS/cm for domestic water use [38]. However, measured values for B1 throughout the investigation were very close to the recommended guideline value of DWAF (Table 1). Hence, frequent monitoring of hotels such as the investigated B1 borehole is required, because this parameter might accumulate overtime and exceeds the recommended level. EC plays an important role in water quality as it gives an indication of salinity and TDS present in water [41]. The total dissolved solids (TDS) that measures the dissolution mechanism of organic and inorganic materials in groundwater were low and below the WHO value of 1000 mg/L. Turbidity recorded (0.33–14.9 NTU) were within the acceptable limit set by DWAF (< 1 NTU) and WHO (< 5 NTU) for domestic water except in April where B3, B4, B5, B6, and B7 exceeded the DWAF limit but fell within the guideline value of WHO for domestic water (Table 1), while B3 (14.9 NTU) and B5 (5.76 NTU) samples during April exceeded both standard limits. Turbidity is caused by colloidal or suspended particles that may originate from organic or inorganic matter or combination of both in water, thus prevents transmission of light through the water. Its affect the appearance and the aesthetic property of water which shows that there is a slight risk of potential secondary health effects turbidity between 1 and 20 NTU and minor risk if used for food preparation [41].

### Anions

Table 2 shows the mean concentration of F, Cl, NO_3_ and SO_4_ in groundwater samples obtained around Muledane village in January, April and June. Fluoride and chloride concentrations ranged from 0.007 to 0.167 mg/L and 4.3 to 46.9 mg/L, respectively. All the boreholes complied with the limit values set by DWAF [38] and WHO [39]. A small concentration of fluoride in the ppb level is needed for good dental health [41]. The highest and lowest concentration of nitrate obtained during the study period was in January and April for B8 and B4 samples with the concentration of 125.18 and 0.6 mg/L (Table 2), respectively. The recommended water quality guideline for nitrate is < 22 and < 50 mg/L by DWAF [38] and WHO [39], respectively. The mean concentration of NO_3_^−^ for all the boreholes during investigation failed to comply with the recommended guideline except for B4 (Table 2). The water samples from the hotel (B1) had higher concentration of nitrate 121.64, 53.129 and 51.55 mg/L in January, April and June, respectively compared to other boreholes. According to DWAF [38], more than 10 mg/L of nitrate may cause methaemoglobinaemia in infants and may also result in the occurrence of mucous membrane irritation in adults if it is more than 20 mg/L.

**Table 2:** Guidelines for drinking water quality set by South Africa and World Health Organisation (WHO)

Heavy metal	Standard limit for drinking water quality in (mg/L) by DWAF [38]	Health based guideline in (mg/L) by WHO [39]
Cadmium	0–0.005^a^	0.003
Copper	0–1	2
Chromium	0–0.05^a,b^	< 0.05
Iron	0–0.1	< 0.3
Lead	0–0.1	0.01
Manganese	0–0.05	< 0.5
Zinc	0–3	< 3

*DWAF* Department of Water Affairs and Forestry, South Africa

^a^Tentative guidelines

^b^For chromium (VI)

### Heavy metal concentration in borehole water

#### Chromium

**Fig. 1:**
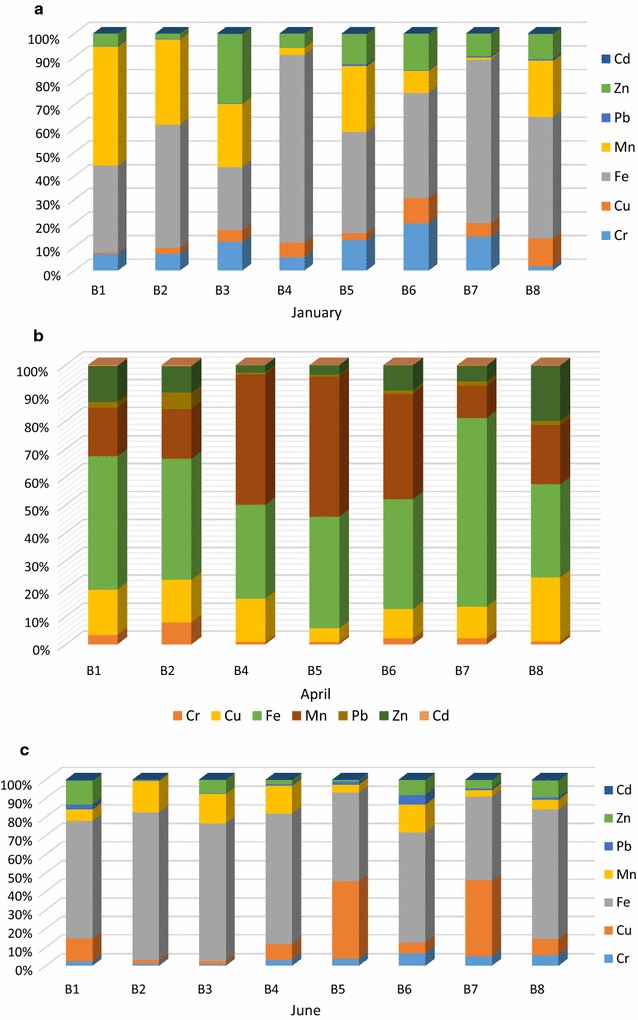
Mean value of physico-chemical parameters in groundwater samples collected from eight boreholes in Muledane village

Figure 1a–c shows the concentration of Fe, Cd, Cr, Zn, Mn, Cu and Pb in the water samples collected from the investigated boreholes at Muledane village during the study period. Chromium concentrations were in the range of 0.005–0.15 mg/L samples for B1–B8 throughout the study. The samples taken from boreholes B1 to B7 in January did not comply with the recommended water quality guidelines of < 0.05 mg/L for both WHO [39] and DWAF [38] for domestic use (Table 2). High Cr concentration in January for these samples could be as results of high infiltration of water and leachates from landfill and dumpsite due to heavy rainfall. Disposal of metal products around this area could have led to high concentration of Cr in the boreholes [42]. According to DWAF [38], consumption of water with Cr concentration greater than 0.05 mg/L has possible risk of inducting gastrointestinal cancer following long-term exposure, undesirable taste and slight nausea in humans. Furthermore, in vitro study has shown that high Cr(III) concentration in the cell could cause DNA damage in humans [43, 44]. It is noteworthy to say that water samples taken from the hotel is very high in NO_3_ and Cr, therefore proper treatment of the water is necessary to make it suitable for the public.

#### Iron

**Fig. 2:**
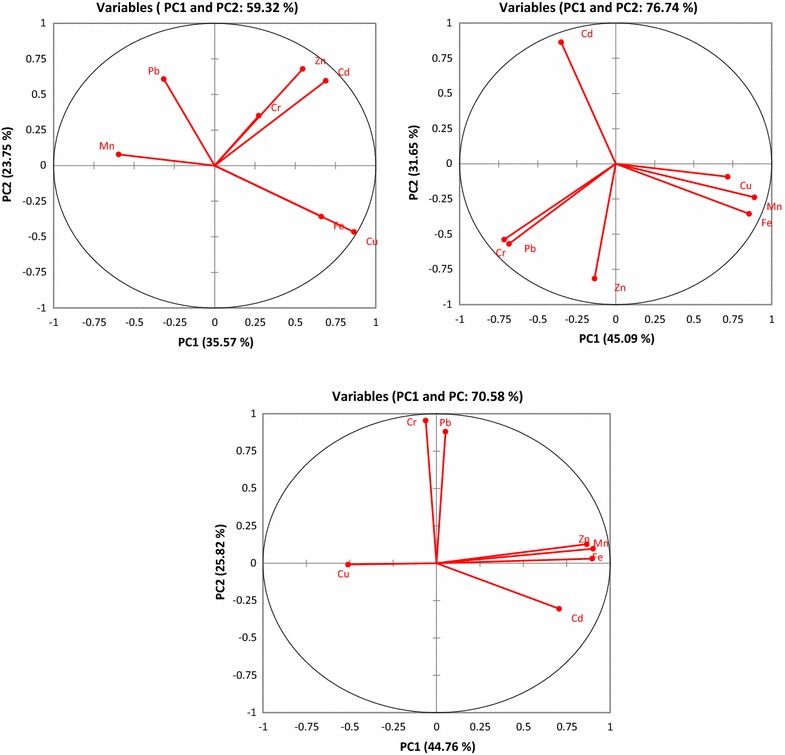
The principal component analysis (PCA) biplots showing the relationships between heavy metals in the borehole samples around Muledane village of Limpopo, South Africa

The mean concentrations of Fe obtained throughout the assessment ranged from 0.15 to 1.86 mg/L (Fig. 1a–c) and were beyond the recommended concentration of < 0.1 and 0.3 mg/L set by DWAF [38] and WHO [45], respectively, for domestic water use. Elevated concentration was observed in January for B4 (1.86 mg/L), B7 (0.72 mg/L) as shown in Fig. 2a; in April B4 (0.88 mg/L), B5 (0.96 mg/L) (Fig. 1b), finally in June, B2 (1.14 mg/L) and B3 (1.45 mg/L, Fig. 1c) were measured. Water with Fe concentration of less than 0.3 mg/L have slight effects on taste and other marginal aesthetic effects such as slight staining of white clothes if used for laundry purposes. However, more than 0.3 mg/L was present in water samples taken from boreholes B4–B7 during the months of January and April. This could result in an adverse aesthetic and health effects when ingested by the residents around Muledane area [33]. High concentration of Fe in Muledane boreholes groundwater could be due to leaching of Fe from the sewer pipes and from other non-point sources such as storm runoff, disposal of metal and municipal landfill. This may also be as a result of nitrate leaching in groundwater, oxidation and decrease in pH could lead to dissolution of iron thus, increases the Fe concentration in groundwater [33, 46].

#### Manganese

The concentrations of Manganese varied from 0.01 to 1.22 mg/L for samples B1–B8 (Fig. 1a–c). All boreholes complied with the WHO [39] guideline concentration of < 0.4 mg/L for domestic water use except for borehole, B1 in January (Fig. 1a) and B4–B7 in April (Fig. 1b). However, all boreholes failed to comply with the standard limit of < 0.05 mg/L set by DWAF [38] for domestic water. This may be as a result of landfill leachates leaching to the boreholes, industrial effluent or indirect contact of water in the boreholes with the sewage. According to DWAF [41], no aesthetic effects associated with the use of water with less than 0.05 mg/L Mn concentration, but concentration between 0.10 and 0.15 mg/L could cause critical stain and taste problems [38, 39].

#### Copper

Average concentration of Cu in all the groundwater samples ranged between 0.01 and 0.41 mg/L (Fig. 1a–c). The concentration is below the standard limits of < 1.0 and < 2 mg/L set by DWAF [38] and WHO [39], respectively for domestic purpose (Table 2). No adverse health effect associated with consumption of water with less than 1.0 mg/L concentration of Cu [41]. Higher concentration was measured at site B4 as compared to other groundwater samples in the month of April (Fig. 1b). The higher concentration could be as a result of Cu particles from the pipes into the borehole water.

#### Lead

The concentrations of Pb ranged between 0.002 and 0.026 mg/L and the mean concentrations in water samples throughout the study period for all the boreholes (B1–B8) are depicted in Fig. 1a–c. Although, the mean value obtained was below the standard guidelines of 0.01 mg/L set by both DWAF [38] and WHO [39] for domestic water use, except sample B6 that exceeded the limits. Specifically, the concentration of B2 (0.026 mg/L) and B3 (0.023 mg/L) in April during autumn and B6 (0.023 mg/L) in June during winter was high (Fig. 1c). Studies have shown that chronic Pb exposure can cause anaemia and high blood pressure especially in older and middle age groups. Exposure to high concentration could cause kidney and brain damage in male [47], while water with less than 0.05 mg/L concentration of Pb could have slight risk of behavioural changes and possibility of neurological impairment in foetuses and young children developing their brain tissues [38].

#### Zinc and cadmium

During the study period, all boreholes complied with the recommended standard limits of < 5.0 and < 3.0 mg/L set for Zn by both WHO and DWAF, respectively for domestic purposes. The maximum and minimum detection values of 0.003 and 0.24 mg/L were recorded in April (B3 sample) and June (B5 sample) as shown in Fig. 1b and c, respectively. The concentration in the collected samples might be due to high water infiltration in April due to rain as compared to other months (Fig. 1). Hence, all boreholes water has little to no health effects because Zn is known to have antioxidant properties that protect humans against accelerated aging of muscles and skin. It’s also helps in healing process after an injury if moderate and recommended dosage is ingested [33]. In addition, the concentration of Cd throughout the study period was below the standard limits set by DWAF [38] and WHO [39] which is 0.005 and 0.003 mg/L, respectively for domestic water use.

### Multivariate analysis

The PCA/FA loading factors for the selected metals in the borehole samples taken around Muledane village for January, April and June are shown in Table 3. Throughout the monitoring period, two important principal components (PCs) were significant with eigenvalues > 1, explaining higher total variance of 59.35, 76.74 and 70.58% for January, April and June, respectively (Table 3 and Fig. 2). In January, two PCs were identified by PCA/FA to be 35.57% (PC1) and 23.75% (PC2) (Fig. 2a). In April, PC1 and PC2 were 45.09 and 31.65% with eigenvalues > 2 (Table 3 and Fig. 2b), while in June, PC1 and PC2 has variables of 44.76 and 22.82% (Fig. 2c), respectively.

**Table 3:** Factor loadings of selected heavy metals in the borehole water samples during the monitoring period

Selected metals	January		April		June	
	PC1	PC2	PC1	PC2	PC1	PC2
Cr	0.274	0.351	− 0.684	− 0.568	− 0.063	0.955
Cu	0.864	− 0.466	0.718	− 0.092	− 0.510	− 0.009
Fe	0.662	− 0.357	0.855	− 0.355	0.897	0.031
Mn	− 0.596	0.079	0.889	− 0.238	0.902	0.097
Pb	− 0.316	0.609	− 0.715	− 0.538	0.052	0.881
Zn	0.546	0.680	− 0.135	− 0.815	0.866	0.127
Cd	0.690	0.596	− 0.350	0.864	0.707	− 0.305
Eigenvalue	2.490	1.662	3.157	2.215	2.821	1.579
Variability (%)	35.568	23.750	45.093	31.648	40.300	22.559
Cumulative %	35.568	59.318	45.093	76.741	40.300	62.859

Pearson correlation showed the inter-relationship between all metals (Table 4). Positive significant correlation of Cu with Fe (R^2^ = 0.734) and Zn (R^2^ = 0.779) were observed in January with weak positive correlation (R^2^ ≥ 0.3) between chromium-iron and cadmium-copper. Copper was negatively correlated with Mn (R^2^ = − 0.633) and Pb (R^2^ = − 0.444). In April, Pb was strongly and positively correlated with Cr (R^2^ = 0.971); Mn with Fe (R^2^ = 0.823) and Cu (R^2^ = 0.710), while strong negative correlation was observed between Cd and Zn (R^2^ = − 0.712). In June, strong relationship among metals was also observed, Fe exhibited relationship with Mn and Zn (R^2^ = 0.995, 0.662, respectively), Pb correlated with Cr (R^2^ = 0.717) while, Zn with Mn, Cd and Fe (R^2^ = 0.662, 0.738 and 0.662, respectively). These metals are likely present in the collected borehole water samples due to agricultural run-off or atmospheric deposition in the study area [36]. In addition, source of heavy metals in the water sample taken from the hotel (B1) could be as a result of linkages from sewage or toilets around the hotel to the groundwater.

**Table 4:** Pearson correlation matrix among metals in the groundwater samples

Variables	Cr	Cu	Fe	Mn	Pb	Zn	Cd
January							
Cr	*1*	0.069	0.371	0.070	0.256	0.199	0.169
Cu	0.069	*1*	*0.734*	− 0.633	− 0.444	0.099	0.307
Fe	0.371	*0.734*	*1*	− 0.041	− 0.231	− 0.064	0.272
Mn	0.070	− 0.633	− 0.041	*1*	0.070	− 0.265	− 0.241
Pb	0.256	− 0.444	− 0.231	0.070	*1*	− 0.066	0.107
Zn	0.199	0.099	− 0.064	− 0.265	− 0.066	*1*	*0.779*
Cd	0.169	0.307	0.272	− 0.241	0.107	*0.779*	*1*
April							
Cr	*1*	− 0.267	− 0.359	− 0.336	*0.971*	0.312	− 0.169
Cu	− 0.267	*1*	0.576	0.710	− 0.245	− 0.124	− 0.142
Fe	− 0.359	0.576	*1*	*0.823*	− 0.386	0.136	− 0.559
Mn	− 0.336	0.710	*0.823*	*1*	− 0.397	− 0.118	− 0.500
Pb	*0.971*	− 0.245	− 0.386	− 0.397	*1*	0.351	− 0.081
Zn	0.312	− 0.124	0.136	− 0.118	0.351	*1*	− 0.712
Cd	− 0.169	− 0.142	− 0.559	− 0.500	− 0.081	− 0.712	*1*
June							
Cr	*1*	0.168	0.019	0.070	*0.717*	0.125	− 0.386
Cu	0.168	*1*	− 0.327	− 0.381	− 0.199	− 0.263	− 0.287
Fe	0.019	− 0.327	*1*	*0.995*	− 0.063	0.662	0.398
Mn	0.070	− 0.381	*0.995*	*1*	0.010	0.663	0.379
Pb	*0.717*	− 0.199	− 0.063	0.010	*1*	0.139	− 0.026
Zn	0.125	− 0.263	0.662	0.663	0.139	*1*	*0.738*
Cd	− 0.386	− 0.287	0.398	0.379	− 0.026	*0.738*	*1*

Values in italic have significance correlation

**Fig. 3:**
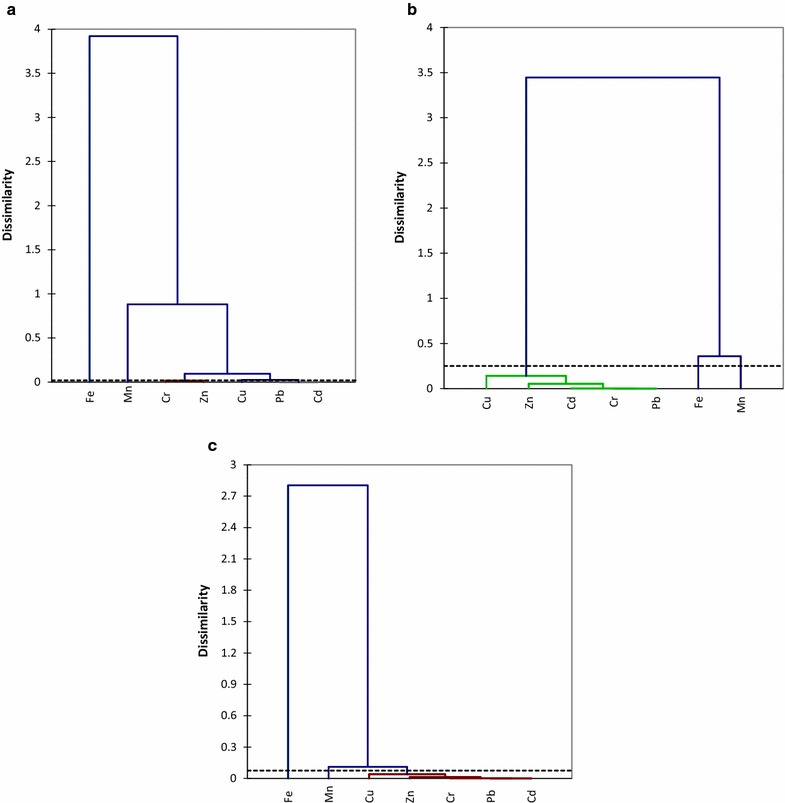
Dendrogram showing the spatial clustering of selected heavy metals in water samples from Muledane boreholes during the monitoring periods based on the hierarchical cluster analysis using Ward’s method

The relationships among the metals were determined by HCA and they were grouped into clusters based on the similarities and dissimilarities between different metals. Dendrogram analysis produced 3 clusters in January and 2 clusters in April and June based on the spatial distribution of metals within these months (Fig. 3). Cluster 1 in January for all samples contained Cr, Zn, Cu, Pb and Cd, cluster 2 include Mn and cluster 3 has Fe (Fig. 3a). Cluster 1 in the dendrogram generated for April is similar with the aforementioned cluster 1, while cluster 2 consists of Fe and Mn (Fig. 3b). In June, cluster 1 has Fe, while 2 is formed by Mn, Cu, Zn, Cr, Pb and Cd (Fig. 3b). The results of cluster analysis supported the correlation results, which suggested that the selected metals are from anthropogenic and natural sources. Occurrence of Mn, Fe, Cd and Zn indicated agricultural and domestic sewage contamination. Run-off of fertilizers or fungicides from the farm, leachates into through the aquifer to the groundwater could also affect water quality [24, 48]. Multivariate analysis using PCA/FA is very useful as a monitoring tools to identify the multiple sources of contaminants and relationships with metals in the groundwater. The PCA and HCA agreed with each other and showed the significant contributions and sources of these metals in groundwater samples. Studies have shown that application of fertilizer during farming are one of the well-known sources of Cd and Cu contamination in groundwater [24, 48].

### Evaluation of human health risk due to heavy metals in groundwater samples

Health risk assessment model by the US. EPA were used to evaluate the health risks that heavy metals could pose on human via direct ingestion and dermal absorption of groundwater in Muledane village. The level of exposure through EX_ing_ and EX_derm_ were estimated for the months of January, April and June. The results suggested that contaminants from the boreholes around Muledane via ingestion and dermal pathways were the major exposure routes to humans in this village. Health related risk associated with the exposure through ingestion depends on the weight, age and volume of groundwater consumed by an individual this was determined using the measured minimum and maximum concentration of Cr, Cd, Zn, Pb, Mn, Fe and Cu.

The hazard quotient (HQ) which is a numeric estimate of the systemic toxicity potential posed by a single element within a single route of exposure was calculated and both HQ_in_ and HQ_derm_ in January, April and June for all the metals were less than one unity (Table 5) for adults and children. This indicates that little or no adverse health effect are likely to be caused by all these metals when the groundwater is consumed or via dermal adsorption by all ages. The HQ_in_ and HQ_derm_ decreased in the order of Cd > Cr > Mn > Zn > Pb > Cu > Fe and Cr > Mn > Zn > Pb > Cd > Cu > Fe > Zn, for both children and adults in January, respectively. HQ_in_ and HQ_derm_ decreased in the order of Mn > Pb > Cr > Cu > Zn > Cd > Fe and Mn > Cr > Pb > Cd > Cu > Fe > Zn, respectively in April, while the order for June were Pb > Mn > Cr > Zn > Cu > Cd > Fe and Cr > Mn > Pb > Cd > Cu > Fe > Zn, respectively for both children and adults. The HQ_Mn_ is the second abundant in January for HQ_derm_ for both pathways in June, while the highest was estimated throughout the pathways in April for all ages, respectively. The results are similar to the findings of Elumalai et al. [24], in which HQ_ing_ for Mn concentration in groundwater for children were higher than one unity. Likewise, Cr that is classified as a known human carcinogenic agent via inhalation is of public health concern. In this study, the highest hazard quotient for Cr through dermal adsorption were observed in January and June, while in April, it has the highest values for both adults and children (Table 5). It has been reported that Cr could originate from different sources either natural or anthropogenic with high environmental mobility [49, 50]. However, it has been suggested that estimated HQ values for metals > 1 for children should not be neglected [51, 52], because children are highly susceptible to pollutants [53]. The main contributors for non-carcinogenic health risk in both pathways were Mn, Pb, Cr and Cd.

**Table 5:** Hazard quotient for potential non-carcinogenic risk (HQ) and cumulative hazard indices (HI) for each heavy metal present in the groundwater samples from the boreholes in Muledane village as consumed by adults and children via ingestion and dermal absorption pathways between January and June

Metals	RfD_ing_ (µg/kg/day)	RfD_derm_ (µg/kg/day)	Statistical parameter	January				April				June			
				Adults		Children		Adults		Children		Adults		Children	
				HQ_ing_	HQ_derm_	HQ_ing_	HQ_derm_	HQ_ing_	HQ_derm_	HQ_ing_	HQ_derm_	HQ_ing_	HQ_derm_	HQ_ing_	HQ_derm_
Cr	3	0.075	Minimum Maximum	8.74E−05 1.51E−03	3.32E−05 5.72E−04	3.33E−04 5.75E−03	2.95E−06 5.09E−05	4.82E−05 3.55E−04	1.83E−05 1.35E−04	1.84E−04 1.35E−03	1.63E−06 1.20E−05	6.13E−05 3.02E−04	2.33E−05 1.15E−04	2.34E−04 1.16E−03	2.07E−06 1.02E−05
Cu	40	8	Minimum Maximum	7.53E−06 1.11E−04	1.79E−07 2.63E−06	2.87E−05 4.23E−04	1.59E−08 2.34E−07	5.17E−05 3.07E−04	1.22E−06 7.28E−06	1.97E−04 1.17E−03	1.09E−07 6.45E−07	1.51E−05 1.37E−04	3.57E−07 3.24E−06	5.75E−05 5.21E−04	3.18E−08 2.88E−07
Fe	700	140	Minimum Maximum	9.47E−06 8.01E−05	2.25E−07 1.90E−06	3.62E−05 3.06E−04	2.00E−08 1.69E−07	6.49E−06 4.15E−05	1.54E−07 9.85E−07	2.48E−05 1.59E−04	1.37E−08 8.76E−08	6.77E−06 6.25E−05	1.61E−07 1.48E−06	2.58E−05 2.39E−04	1.43E−08 1.32E−07
Mn	24	0.96	Minimum Maximum	1.26E−05 1.08E−03	1.49E−06 1.28E−04	4.80E−05 4.12E−03	1.32E−07 1.14E−05	1.00E−04 1.53E−03	1.19E−05 1.81E−04	3.82E−04 5.83E−03	1.06E−06 1.61E−05	1.49E−05 3.91E−04	1.77E−06 4.64E−05	5.71E−05 1.49E−03	1.58E−07 4.13E−06
Pb	1.4	0.42	Minimum Maximum	3.44E−05 1.85E−04	2.18E−06 1.17E−05	1.32E−04 7.07E−04	1.94E−07 1.04E−06	1.46E−04 5.64E−04	9.26E−06 3.57E−05	5.59E−04 2.15E−03	8.23E−07 3.17E−06	5.81E−05 4.97E−04	3.68E−06 3.15E−05	2.22E−04 1.90−03	3.27E−07 2.80E−06
Zn	30	60	Minimum Maximum	4.02E−05 2.41E−04	5.72E−08 3.43E−07	1.53E−04 9.21E−04	5.09E−09 3.05E−08	4.26E−05 2.34E−04	6.06E−08 3.34E−07	1.63E−04 8.95E−04	5.39E−09 2.97E−08	2.81E−06 1.43E−04	4.00E−09 2.03E−07	1.07E−05 5.45E−04	3.56E−10 1.81E−08
Cd	0.5	0.025	Minimum Maximum	6.03E−05 2.41E−03	5.72E−06 1.14E−05	4.60E−03 9.21E−03	5.09E−07 1.02E−06	6.03E−05 1.21E−04	5.72E−06 1.14E−05	2.30E−04 4.60E−04	5.09E−07 1.02E−06	6.03E−05 9.04E−05	5.72E−06 8.58E−06	2.30E−04 3.45E−04	5.09E−07 7.63E−07
HI_*ing*/derm_	–	–	Minimum Maximum	2.52E−03 5.61E−03	4.31E−05 7.28E−04	5.33E−03 2.14E−02^a^	3.83E−06 6.7E−05	4.56E−04 3.15E−03	4.66E−05 3.72E−04	1.74E−03 1.20E−02^a^	4.14E−06 3.30E−05	2.19E−04 1.62E−03	3.50E−05 2.06E−04	8.37E−04 6.20E−03	3.11E−06 1.83E−05

^a^Calculated maximum HI values found in the sample

The calculated cumulative hazard quotients (HI) across metal served as a conservative assessment tool to estimate high-end risk rather than low end-risk in order to protect the public (Table 5). This served as a screen value to determine whether there is major significant health risk that exposure of heavy metals in the groundwater may pose on the villagers and if there is any difference in total health risk during the study period. The estimated total HQ values were less than one (Table 5), therefore, exposure to these elements through mouth ingestion and dermal adsorption through the skin may likely not exert negative or cumulative adverse risk on the inhabitants of this village.

The average estimated minimum and maximum values for chronic daily intake (CDI) for the selected heavy metals in groundwater samples collected from the boreholes around Muledane via ingestion pathway for both adults and children are shown in Table 6. The maximum CDI values for the selected metals in January, April and June ranged between 5.85E−02–4.17E−05, 3.82E−02–6.29E−05 and 4.56E−02–4.17E−05 for adults, while children index was 2.23E−01–2.40E−04, 1.46E−01–2.40E−04 and 1.74E−01–1.80E−04, respectively. The CDI indices for heavy metals during the study period for both ages were found to be in the order of Fe > Mn > Zn > Cr > Cu > Pb > Cd in January; Mn > Fe > Cu > Zn > Cr > Pb > Cd in April and finally Fe > Mn > Cu > Zn > Cr > Pb > Cd in June (Table 6). In the drinking water of Muledane groundwater, high CDI values of Mn, Fe and Cu were estimated for both adults and children, also high estimated values for children ingesting Zn were observed throughout the study. Wu et al. [35] and Naveedullah et al. [34] suggested that high Zn, Mn and Fe are from agricultural practices such as run-off from extensive farming area, use of fungicides and fertilizers affect water quality. In general, health risk assessment index using the overall non-carcinogenic risk assessment (HI), CDI and HQ via ingestion and dermal adsorption routes were less than one unity. This is an indication that groundwater poses less significant health threats to both adults and children via the pathways [33, 35], however measures should be made to avoid accumulation of heavy metals that could pose any health problems especially in children.

**Table 6:** Chronic risk assessment (CDI_ing_) of heavy metals in groundwater samples taken around Muledane village through daily ingestion pathway during January, April and June for adults and children

Metals	January		April		June	
	Adults	Children	Adults	Children	Adults	Children
Cr	2.73E−04–4.71E−03	1.04E−03–1.80E−02	1.51E−04–1.11E−03	5.76E−04–4.24E−03	1.92E−04–9.46E−04	7.30E−04–3.61E−03
Cu	3.144E−04–4.62E03	1.20E−03–1.76E−02	2.16E−03–1.28E−02	8.23E−03–4.89E−02	6.29E−04–5.69E−03	2.40E−03–2.17E−02
Fe	6.91E−03–5.85E−02	2.64E−02–2.23E−01	4.74E−03–3.03E−02	1.81E−02–1.16E−01	4.94E−03–4.56E−02	1.89E−02–1.74E−01
Mn	3.14E−04–2.70E−02	1.20E−03–1.03E−01	2.50E−03–3.82E−02	9.56E−03–1.46E−01	3.74E−04–9.80E−03	1.43E−03–3.74E−02
Pb	5.03E−05–2.70E−04	1.92E−04–1.03E−03	2.14E−04–8.23E−04	8.16E−04–3.14E−03	8.49E−05–7.26E−04	3.24E−04–2.77E−03
Zn	1.26E−03–7.54E−03	4.80E−03–2.88E−02	1.33E−03–7.34E−03	5.09E−03–2.80E−02	8.80E−05–4.46E−03	3.36E−04–1.70E−02
Cd	3.14E−05–6.29E−05	1.20E−04–2.40E−04	3.14E−05–6.29E−05	1.20E−04–2.40E−04	3.14E−05–4.17E−05	1.20E−04–1.80E−04

Carcinogenic risk (CR_ing_) defined as the incremental probability that an individual will develop cancer during one’s lifetime due to exposure under specific scenarios were calculated for the selected metals in this study [35]. Only carcinogenic risk of Cr, Pb and Cd for Muledane groundwater were calculated for both adults and children, because the value of carcinogenic slope factor for Cu, Fe, Mn and Zn could not be found in the literature. The maximum estimated CR_ing_ values are shown in Table 7. Throughout the study, the average levels of CR_ing_ for Pb ranged between 3.05E−05–9.29E−05 for adults and 1.16E−04–3.55E−04 for children. In general, under most regulatory program the carcinogenic risk values between 10^−6^ and 10^−4^ for an individual suggest potential risk, hence the results in this study suggested that the level of Cr and Pb in the groundwater could pose carcinogenic risk to both adults and children. Therefore, proper control measures to protect the health of humans around the study area should be put in place in order to ensure safety of consumers. Also, concerted efforts are required for sustainability of the groundwater by removing these metals.

**Table 7:** Carcinogenic risk assessment (CR_ing_) of Cr, Pb and Cd at different times of groundwater samples collected around Muledane village through ingestion pathway for adults and children between January, April and June

Metals	January		April		June	
	Adults	Children	Adults	Children	Adults	Children
Cr	5.24E−07–9.04E−06	2.00E−06–3.45E−05	2.89E−07–2.13E−06	1.10E−06–8.12E−06	3.68E−07–1.81E−06	1.40E−06–6.93E−06
Pb	5.67E−06–3.05E−05	2.17E−05–1.16E−04	2.41E−05–9.29E−05	9.21E−05–3.55E−04	9.57E−06–8.19E−05	3.66E−05–3.13E−04
Cd	4.94E−09–9.88E−09	1.89E−08–3.77E−08	4.94E−09–9.88E−09	1.89E−08–3.77E−08	4.94E−09–7.41E−09	1.89E−08–2.83E−08

## Conclusions

Only 12.5% boreholes have ideal water quality in terms of NO_3_^−^ and Mn concentration with 25% found to be in the marginal water quality class, while 75% percent fell in the unacceptable water quality class. In terms of chemical properties, it is unsafe for resident around Muledane within the investigated area to use the boreholes water for domestic purposes without treatment. This study reveals that 87.5% borehole water have high concentration of NO_3_; Fe and Mn among the selected anions and heavy metals. The measured concentration of Cr, Fe and Mn for some of the investigated boreholes were observed to be higher than the recommended standard limits by WHO and DWAF. The HQ and the overall non-carcinogenic health hazard indices (HI) through the ingestion and dermal adsorption of the groundwater were less than one. However, the results showed the potential risk of some of the selected metals on human, especially children. The main contributors to non-carcinogenic risk were Mn, Zn, Pb, Cr and Cd for both pathways. The results of this study further revealed that ingestion of the investigated boreholes poses carcinogenic risk (CR_ing_) regarding the estimated Mn, Fe and Cu for adults and children. In addition to the aforementioned metals, estimated CR_ing_ for Zn among children were high throughout the study. It is therefore recommended that water quality studies should be given a priority by adding it into the integrated development plans (IDPs) and be conducted on a regular basis to assess risks of contamination. Health and hygiene education is highly needed for people in rural areas because of lack of proper sanitation and proper water handling practices. In addition, further studies are recommended to investigate the point sources of contamination and possible causes of high concentration of nitrate level in the boreholes around Muledane village.
